# Environmental and Health Risk Assessments of Antibiotics and Heavy Metals in Manure in Liuyang City

**DOI:** 10.3390/toxics14030201

**Published:** 2026-02-27

**Authors:** Yuli Jiang, Ziwen Guo, Manjun Miao, Xueduan Liu, Luhua Jiang

**Affiliations:** 1 School of Minerals Processing and Bioengineering, Central South University, Changsha 410083, China; 2 Key Laboratory of Biometallurgy of Ministry of Education, Central South University, Changsha 410083, China

**Keywords:** manure, combined pollution, manure microbial community, microbial function prediction, risk assessment, Monte Carlo simulation

## Abstract

With the rapid growth of livestock farming, the use of antibiotics and heavy metals as feed additives has raised environmental and health concerns. This study systematically analyzed the contamination levels of antibiotics and heavy metals in manure samples collected from five farms of varying scales in Liuyang City, Hunan Province. Utilizing 16S rDNA high-throughput sequencing technology, the study also examined the structural and functional characteristics of manure microbial communities. Ecological risk and human health risk assessments were also conducted. Results revealed that antibiotic residues in pig manure were generally higher than those in chicken manure, with significant differences in antibiotic usage across farms of varying scales. Cu and Zn levels exceeded standards in some samples, particularly from small-scale farms. Microbial community structures showed marked differences, with pig manure exhibiting higher microbial diversity. Functional prediction indicated active metabolism, strong environmental adaptability, and robust pollutant degradation capacity. Risk assessments revealed moderate to high ecological and human health risks from certain antibiotics and heavy metals, with significant non-carcinogenic and carcinogenic risks particularly for children. The study emphasizes that rational control of antibiotic and heavy metal use, coupled with enhanced manure management and resource utilization, is crucial for safeguarding ecological security and public health.

## 1. Introduction

To prevent and control infectious diseases in livestock and poultry, protect animal health, improve feed utilization efficiency, and reduce farming costs, feed additives such as antibiotics and heavy metals are extensively used in the livestock and poultry farming industry. According to statistics, over 60% of antibiotics used annually in the United States are veterinary antibiotics [1]. The total amount of antibiotics used per kilogram of animal in Europe ranges from approximately 20 to 180 milligrams. In Asia, veterinary antibiotic usage is even higher, with the average antibiotic use per kilogram of live poultry being about two to three times that of Europe [2]. China is a major producer and consumer of livestock and poultry, as well as a major producer and user of veterinary antibiotics. Statistics indicate that China launched an initiative to reduce the use of veterinary antibiotics starting in 2018. In 2018, the total amount of antibiotics used in China’s livestock, poultry, and aquaculture industries was approximately 30,000 tons. The total amount of antibiotics used per ton of animal products produced in China was about 140 g. Although the usage of veterinary antibiotics has decreased compared to before the reduction initiative, the total consumption of veterinary antibiotics remains higher than the standards set by developed countries [3]. Antibiotics and heavy metals remaining in livestock and poultry manure can be partially removed through solid waste disposal and biodegradation during organic fertilizer production, but they cannot be eliminated [4]. These residues have become a major source of soil and water pollution, posing risks to the ecological environment and public health. In particular, the spread of antibiotic resistance genes has drawn increasing attention [5,6].

In the livestock and poultry farming industry, heavy metals are commonly used as feed additives in combination with antibiotics. Cu, Zn, Cr, and As are among the most frequently utilized heavy metals in animal husbandry, with Cu and Zn being the most widely employed and present in the highest concentrations [7]. Heavy metal additives can enhance the immunity of livestock and poultry, improve coat color, increase feed efficiency, and promote animal growth and development. The livestock and poultry farming industry frequently abuses heavy metal additives in pursuit of higher economic returns. The absorption rate of heavy metals in animal intestines is extremely low, with over 95% of heavy metals being excreted from the animal’s body and accumulating in their waste. Unlike antibiotic residues, heavy metals are not biodegradable by microorganisms. Consequently, heavy metals exhibit an accumulation effect in the environment, leading to manure becoming a significant reservoir for heavy metals [8].

Liuyang City in Hunan Province, as a major region for livestock and poultry farming, boasts a long history of animal husbandry and a high farming density. Since the late 1980s, the Liuyang Municipal Party Committee and Municipal Government have promoted local economic development by encouraging farmers to engage in animal husbandry through supportive policies, providing technical guidance, and offering preferential treatment to large-scale breeders. Moreover, the steady improvement in people’s living standards and the expanding market demand for poultry and livestock meat have spurred the rapid development of the livestock industry in Liuyang. Consequently, the income and living standards of local villagers have also seen widespread improvement. However, as the number and scale of livestock farms increase, the volume of manure and wastewater generated continues to rise, while the environment’s capacity to absorb these pollutants diminishes. The antibiotics and heavy metals remaining in manure pose a serious threat to the environment. Heavy metals and antibiotics are not only difficult for microorganisms to degrade but may also promote the spread of resistance genes, leading to ecosystem imbalance and environmental pollution [3,9]. Therefore, conducting a systematic assessment of the environmental risks posed by antibiotics and heavy metals in manure is crucial for guiding the sustainable development of the livestock industry and environmental protection.

Therefore, this study conducted manure collection and testing at five livestock farms of varying scales in Liuyang City, Hunan Province. It identified the types and contamination levels of heavy metals and antibiotics in the manure, and performed ecological risk assessments and human health risk assessments.

## 2. Materials and Methods

### 2.1. Manure Samples

**Figure 1:**
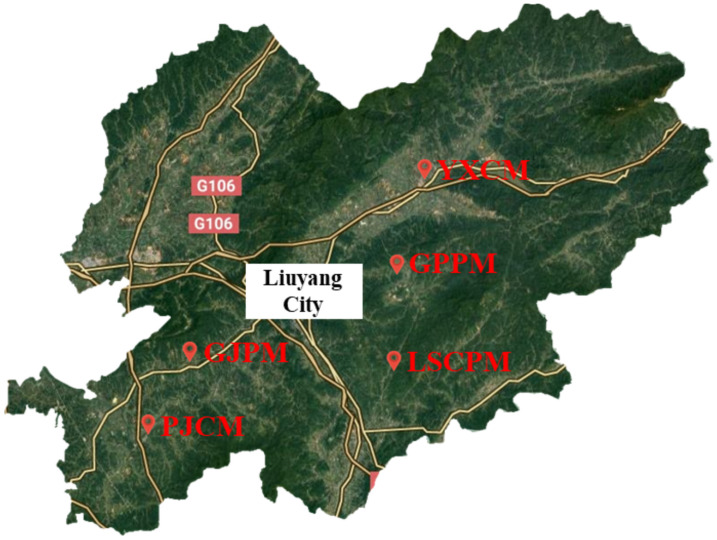
Manure sampling sites in Liuyang City.

This study collected fresh manure samples from five farms of varying scales located in Liuyang City, Hunan Province (Figure 1). Due to practical constraints such as access permissions and feasibility of collaboration, the number of farms available for sampling was limited. Among the accessible farms, the five selected were chosen for their strong representativeness in terms of scale and management practices. These farms range from small-scale to large-scale operations and are geographically well distributed across Liuyang City, enabling a comprehensive reflection of the region and ensuring representativeness. At each farm, multiple subsamples were systematically collected from different locations within the manure storage or pile. These subsamples were thoroughly mixed to form a single composite sample per farm. The composite samples were then placed in resealable bags, transported on ice to the laboratory, and stored at 4 °C until further analysis. Specific sample numbers, sampling locations, and sample types are detailed in Table S1.

### 2.2. Antibiotic and Heavy Metal Concentration Determination in Manure

Total antibiotics in manure were determined using solid-phase extraction coupled with liquid chromatography–mass spectrometry (SPE-LC-MS/MS) [10]. The sample was first dried in a freeze dryer. Then, 5 g of the dried sample was weighed and placed into a 50 mL centrifuge tube, and 0.4 g of Na_2_EDTA (Sinopharm Chemical Reagent Co., Ltd., Shanghai, China) was added. Next, 10 mL of an acetonitrile–phosphate buffer solution (volume ratio = 1:1) was added to each tube. The mixture was shaken manually for 2 min, sonicated for 20 min, and then centrifuged at 3500 rpm for 10 min. The supernatant was collected. The residue was extracted twice more, and all supernatants were combined to obtain a total volume of 30 mL. The combined supernatant was concentrated to 15 mL using a parallel evaporator. This concentrated solution was transferred to a 40 mL amber glass vial, and 15 mL of ultrapure water was added. To remove impurities, the solution was purified by passing it through an SAX anion exchange cartridge (200 mg/6 mL) (Agilent Technologies, San Jose, CA, USA). The filtrate was collected and concentrated using an HLB solid-phase extraction cartridge (Agilent Technologies, San Jose, CA, USA) for enrichment. The extraction cartridge was activated sequentially with 5 mL of methanol, 5 mL of deionized water, and 5 mL of pH 2.0 deionized water. When approximately 2 mL of the pH 2.0 deionized water remained above the sorbent, the entire sample was passed through the cartridge at a flow rate of 10–15 mL/min. The cartridge was then washed with 5 mL of deionized water. Subsequently, it was dried under a nitrogen stream for 20 min. Finally, the analytes were eluted with 10 mL of methanol at a flow rate of 5 mL/min, and the eluent was collected. The eluent was concentrated to near dryness using a nitrogen evaporator, diluted to a final volume of 1.0 mL, filtered through a 0.22 μm organic-solvent-compatible membrane, and then stored for subsequent analysis. The instrument model used is LCMS8050 (Shimadzu, Shanghai China), with a column model of InertSustain AQ-C18 1.9 μm 2.1 mm × 50 mm (up) (GL Sciences, Shanghai, China). Detailed parameters for LCMS are shown in Table S2, and the elution program is shown in Table S3.

Total heavy metal content in manure was determined and quantitatively analyzed using microwave digestion coupled with inductively coupled plasma mass spectrometry (ICP-MS) [11]. 0.2000 g of the freeze-dried and sieved sample was weighed into a digestion tube. Inside a fume hood, 5 mL of nitric acid, 3 mL of hydrochloric acid, and 2 mL of hydrofluoric acid were sequentially added to the tube. The sample and the digestion acids were then thoroughly mixed. Subsequently, microwave digestion was performed according to the heating program detailed in Table S4. Following the completion of digestion, the cap of the digestion tube was rinsed with a small amount of deionized water, and the rinse solution was poured back into the tube. The digestion tubes were placed in an acid-evaporation apparatus, and the solution was evaporated at 160 °C. When the liquid became viscous, the tube was removed and allowed to cool slightly. A small amount of dilute aqua regia (1:1) was added, and the mixture was heated to dissolve the residue. The resulting solution was transferred quantitatively into a 25 mL volumetric flask. The flask was then filled to the mark with diluent and mixed thoroughly. Finally, the concentrations of the heavy metals were determined using ICP-MS.

Heavy metal speciation was determined using modified European Bureau Community of Reference (BCR). Through this sequential extraction process with specific chemical reagents, the heavy metals were operationally categorized into four fractions: the acid-extractable fraction (F1), the reducible fraction (F2), the oxidizable fraction (F3), and the residual fraction (F4) [12].

### 2.3. High-Throughput Sequencing Analysis of 16S rDNA

Microbial genomic DNA extraction from livestock and poultry manure was performed using the E.Z.N.A.^®^ Soil DNA Kit (DP712-02) (Tiangen, Beijing, China). Targeted amplification of the V3-V4 hypervariable region of the 16S rDNA gene was performed using universal primers 341F (5′-CCTACGGGNGGCWGCAG-3′) and 805R (5′-GACTACHVGGGTATCTAATCC-3′). Specific PCR reaction conditions and component configurations are detailed in Tables S5 and S6. Amplified products were purified using AMPure XT Magnetic Beads (Beckman Coulter Genomics, Danvers, MA, USA) and quantified using the Qubit^®^ Fluorometric Quantification System (Invitrogen, Waltham, MA, USA). During library preparation, purified DNA fragments were quality-assessed using the Agilent 2100 Bioanalyzer system (Agilent Technologies, San Jose, CA, USA) with the Illumina^®^ Library Quantification Kit (Kapa Bio sciences, Woburn, MA, USA). Qualified library concentrations must be ≥2 nM. After specific index labeling, samples underwent gradient dilution and equimolar pooling according to sequencing requirements. Following alkaline denaturation, single-stranded DNA templates were prepared. Final sequencing analysis was performed using the Illumina NovaSeq 6000 platform (Illumina, San Diego, CA, USA) with the SP Reagent Kit (500 cycles) for paired-end 250 bp read length analysis.

### 2.4. Ecological Risk Assessment Methods

#### 2.4.1. Risk Quotient of Antibiotics

The ecological risk of antibiotics in the environment can be assessed using the risk quotient (RQ) value. RQ is generally derived from the ratio of the measured environmental concentration (MEC) to the predicted no-effect concentration (PNEC), through Equation (1) [13].

(1) $$RQ=\frac{MEC}{PNEC}$$

RQ values are categorized into four levels: RQ < 0.01 (insignificant risk), 0.01 ≤ RQ < 0.1 (low risk), 0.1 ≤ RQ < 1 (medium risk), and RQ ≥ 1 (high risk) [13].

#### 2.4.2. Risk Index of Heavy Metals

The potential ecological risk index (PERI) comprehensively considers factors such as pollutant concentration, toxicity, background values, and ecosystem sensitivity to pollutants. It quantifies the potential ecological risk of pollutants to evaluate their potential ecological risk level [14]. This method reflects not only the environmental impact of a single pollutant element but also the combined effects of multiple pollutants. The calculation formulas are as follows:

(2) $$P_{i}=\frac{C_{i}}{B_{i}}$$

(3) $$E_{i}=T_{i}\times P_{i}$$

(4) $$RI=\sum E_{i}$$

$P_{i}$ denotes the pollution index for heavy metal i, $C_{i}$ represents the measured concentration of heavy metal i, and $B_{i}$ indicates the background values of heavy metal i. The arithmetic mean of Hunan Province’s soil heavy metal background values was adopted as the background value (Table S7). $E_{i}$ signifies the potential ecological risk index for individual heavy metal i, while $T_{i}$ denotes the toxicity response coefficient for heavy metal i, with values set as: As = 10, Cd = 30, Cr = 2, Cu = Ni = Pb = 5, Zn = 1 [14]. RI is a comprehensive potential ecological risk index for multiple heavy metals. Its classification criteria are shown in Table S8.

#### 2.4.3. Risk Assessment Code of Heavy Metals

Risk assessment based on the geochemical fractionation of heavy metals can more accurately reflect the bioavailability of heavy metals in sediments and their potential ecological risks. Risk assessment code (RAC) is a commonly used metric for evaluating the ecological risk posed by the soluble chemical forms of heavy metals [14]. RAC is defined as the proportion of acid-soluble components relative to the total heavy metal content, calculated using the following formula:

(5) $$RAC=\frac{C_{iF1}}{C_{i}}\times 100\%$$

$C_{iF1}$ (mg/kg) represents the acid-extractable heavy metal content determined via the BCR sequential extraction method, indicating the readily bioavailable fraction; $C_{i}$ (mg/kg) denotes the measured concentration of heavy metal i. Based on risk assessment code values, ecological risks of heavy metals are classified into five tiers, with specific classification criteria detailed in Table S9 [14,15].

#### 2.4.4. Health Risk Assessment Methods for Heavy Metals

The health risk assessment was conducted according to the methodology of the United States Environmental Protection Agency (USEPA) to perform a quantitative analysis of the risks posed by soil heavy metals to different population groups. To ensure accurate assessment, the evaluation considered adults and children as separate demographic groups. It covered three primary exposure pathways: direct ingestion ($D_{ing}$), dermal contact ($D_{derm}$) and oral and nasal inhalation ($D_{inh}$) [16]. The formulas for calculating the average daily dose (ADD) for the three exposure pathways ware as follows:

(6) $${ADD}_{ing}=\frac{C_{i}\times R_{ing}\times EF\times ED}{BW\times AT}\times 10^{-6}$$

(7) $${ADD}_{derm}=\frac{C_{i}\times SA\times SL\times ABF\times EF\times ED}{BW\times AT}\times 10^{-6}$$

(8) $${ADD}_{inh}=\frac{C_{i}\times R_{inh}\times EF\times ED}{PEF\times BW\times AT}$$

${ADD}_{ing}$, ${ADD}_{derm}$ and ${ADD}_{inh}$ represent the daily average intake of heavy metals via direct ingestion, dermal contact and oral and nasal inhalation; $C_{i}$ denotes the measured concentration of heavy metal i. Other parameter values and their distribution types are shown in Table S10 [17].

Health risks were categorized into carcinogenic risks and non-carcinogenic risks. Non-carcinogenic risks described the potential non-cancerous health impacts of specific heavy metals, assessing whether exposure levels could cause adverse health effects. Carcinogenic risks quantified the probability of an exposed population developing cancer over a lifetime due to contact with a carcinogen under specific exposure conditions. The respective formulas are provided below:

(9) $$HI=\sum HQ=\sum \frac{{ADD}_{ij}}{{RfD}_{ij}}$$

(10) $$TCR=\sum CR=\sum {ADD}_{ij}\times {SF}_{ij}$$

HI and HQ represent the combined and single non-carcinogenic risk indices, respectively. TCR and CR denote the combined and single carcinogenic risk indices, respectively. ${ADD}_{ij}$ is the daily average exposure dose for non-carcinogenic and carcinogenic effects of heavy metal i via exposure pathway j. ${RfD}_{ij}$ is the corresponding reference dose of heavy metal i via exposure pathway j. ${SF}_{ij}$ is the corresponding slope factor of heavy metal i via exposure pathway j. Specific reference values are provided in Table S11 [17]. According to the USEPA guidelines, the risk threshold for non-carcinogenic effects was set at 1. When HI or HQ < 1, the non-carcinogenic risk is negligible; otherwise, a non-carcinogenic risk exists. When TCR or CR < 1 × 10^−6^, carcinogenic risk is negligible; When 1 × 10^−6^ ≤ TCR or CR ≤ 1 × 10^−4^, carcinogenic risk is cautionary; When TCR or CR > 1 × 10^−4^, carcinogenic risk is unacceptable.

#### 2.4.5. Monte Carlo Simulation

The Monte Carlo simulation method introduced probability distributions for fixed parameters, such as environmental factors, age, body weight, intake rates, and metabolism, into health risk assessment models. It converted deterministic sampling into random sampling and performed numerous iterative simulations, which effectively reduced the likelihood that assessment results would deviate from reality during the risk evaluation process [18]. In contrast to conventional health risk assessment models that rely on fixed parameters for calculation, Monte Carlo simulations utilized a large number of randomly generated numbers conforming to defined probability distributions as input parameters. This approach produced probability distributions for the output (predicted) variables [17]. In this study, Monte Carlo simulation was integrated with health risk assessment using Oracle Crystal Ball software (version 11.1) to quantify uncertainties in the model. By performing 10,000 simulation iterations at a 95% confidence level, the method achieved more precise simulation results and brought the health risk assessment outcomes closer to real-world conditions [19,20].

#### 2.4.6. Data Processing and Statistical Analysis

Statistical analyses were conducted using Microsoft 365 (Excel). During sequencing data processing, paired-end reads were first demultiplexed based on barcode information, with adapter and barcode sequences removed. The resulting split data were then merged and subjected to quality filtering to remove low-quality reads and chimeric sequences, thereby improving data reliability. Following preprocessing, the DADA2 algorithm was applied for length filtering and denoising. By accurately modeling sequencing error rates, DADA2 effectively differentiates true biological sequences from sequencing errors, producing high-quality Amplicon Sequence Variants (ASVs) and corresponding abundance tables. To further ensure data accuracy, singleton ASVs—those with a total sequence count of one across all samples—were removed as a default step, since such low-abundance sequences are often artifacts or contaminants. Subsequent diversity analyses were conducted based on the curated ASV feature sequences and abundance data. Principal Coordinate Analysis (PCoA) based on the Bray–Curtis dissimilarity coefficient was performed using the ade4 package (version 1.7.13) in the R platform (version 4.2.3). Functional pathway annotation was performed using PICRUSt2, referencing the Kyoto Encyclopedia of Genes and Genomes (KEGG) database, with relative abundances obtained for pathway levels 1, 2, and 3. One-way analysis of variance (ANOVA) was used to test for differences among treatments, followed by Tukey’s HSD test for multiple comparisons (*p* < 0.05), using SPSS v28.0 (IBM Corp., Armonk, NY, USA). Other graphs were plotted by Origin 2026.

## 3. Results and Discussion

### 3.1. Antibiotic Residues in Manure

**Figure 2:**
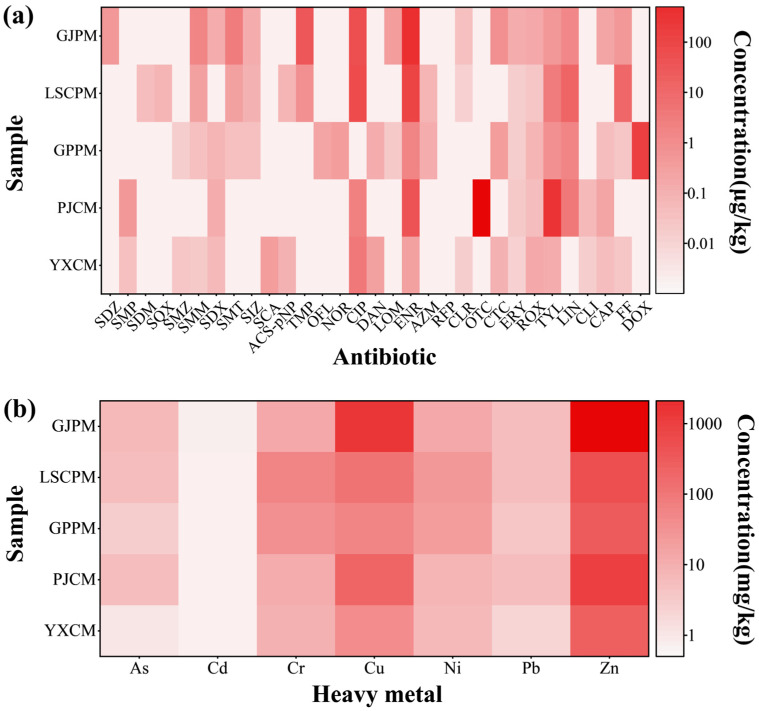
Antibiotics (**a**) and heavy metals (**b**) in manure.

Multiple antibiotic residues were detected across all five manure samples (Figure 2a, Table S12), reflecting widespread antibiotic use in regional agriculture. Overall, pig farm manure contained significantly higher antibiotic levels than chicken farms, with quinolones such as enrofloxacin (ENR) and ciprofloxacin (CIP) particularly prominent. Notably, the small-scale pig farm GJPM exhibited an exceptionally high ENR residue (457.568 μg/kg), indicating intensive use in swine disease control. Tetracycline antibiotics showed site-specific patterns, oxytetracycline (OTC) was found only in chicken farm PJCM at high levels (824.665 μg/kg), while doxycycline (DOX) dominated in large-scale pig farm GPPM, reflecting distinct usage practices. Macrolides like tylosin (TYL) and lincomycin (LIN) were present in both chicken and pig farms, highlighting their widespread application. Sulfonamides showed low detection rates, likely due to regulatory restrictions or replacement by other drugs. Among chicken farms, the larger-scale YXCM had lower antibiotic residues than smaller PJCM, suggesting that farm-specific management rather than scale drives antibiotic use. Pig farms displayed a more complex pattern: the smallest GJPM had highest quinolones and trimethoprim residues; medium-scale LSCPM had florfenicol and LIN residues; and the largest GPPM showed elevated tetracyclines. This nonlinear pattern indicates that antibiotic residues are influenced by multiple factors including scale, disease pressure, and medication practices, with smaller farms potentially prone to misuse due to technical limitations.

Regarding environmental risks, the direct application of untreated manure to farmland could lead to the accumulation of high concentrations of antibiotics, such as ENR, CIP, and OTC, in soil and water bodies. This accumulation could promote the spread of resistance genes, threatening ecosystem stability and public health safety [1,21]. Research indicates that antibiotic residues alter soil microbial communities, affecting nutrient cycling and ecological functions, and may even be transmitted to humans through the food chain [22]. Therefore, rational control of antibiotic use in livestock farming and improvement of manure treatment and resource utilization technologies have become key measures to reduce environmental antibiotic pollution and resistance risks. In summary, this study reveals the diversity and complexity of antibiotic residues in manure in the Liuyang region of Hunan Province. It highlights the impact of farming scales, management practices, and medication habits on antibiotic residue levels. Furthermore, it urges relevant authorities to strengthen oversight of antibiotic use and environmental risk assessments, thereby promoting green farming and sustainable development.

### 3.2. Heavy Metal Residues in Manure

Test results for heavy metal residues in manure (as shown in Figure 2b and Table S12) indicate that the As, Cd, Cr, Ni, and Pb content in all samples did not exceed the limits specified in the Organic Fertilizer Standard (NY/T 525-2021) or the German reference limits for matured compost. This demonstrates that the accumulation risk of these heavy metals is generally controllable within the current sampling system [23]. Hg was not detected or was below the detection limit in all samples, indicating a low risk of Hg contamination from manure in this area. However, the concentrations of Cu and Zn in some samples were significantly higher than the standard limits. In particular, the Cu content in the pig manure sample GJPM reached 1300 mg/kg, far exceeding the reference limit of 100 mg/kg, while its Zn content reached 2100 mg/kg, significantly surpassing the limit of 400 mg/kg. The chicken manure sample PJPM also showed a Zn content of 1000 mg/kg, exceeding the standard limit by 2.5 times. This suggests that the accumulation of Cu and Zn may be related to their historical or current use in feed additives. In terms of farm scale and livestock type, overall Cu and Zn levels in pig manure were generally higher than in chicken manure. Significant variations existed among pig farms of different scales, with the smallest-scale GJPM samples exhibiting the highest Cu and Zn concentrations. Chicken farms exhibited similar characteristics. The smaller-scale PJCM sample showed significantly elevated Zn levels (1000 mg/kg), while the larger-scale YXCM sample met standards (250 mg/kg). This discrepancy may be attributed to certain irregularities in feed formulation, manure management, and pollution control practices at smaller-scale farms. Although most heavy metal levels remain within safe limits, the significant accumulation of Cu and Zn in certain manure samples warrants attention. Particular vigilance is required when utilizing such materials as organic fertilizers, necessitating enhanced monitoring and control measures to prevent potential risks to soil environments and agricultural product safety [3].

**Figure 3:**
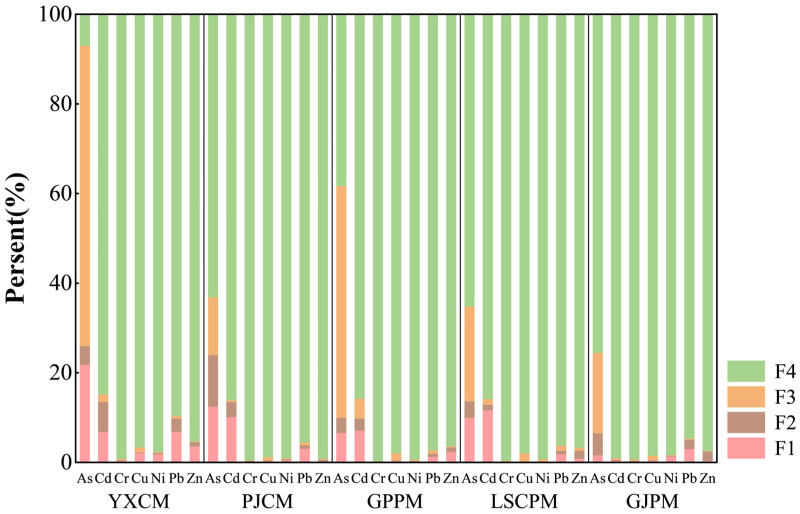
Distribution of heavy metal fractions in manure.

As shown in Figure 3, the analysis of heavy metal speciation in manure samples reveals that the distribution of heavy metal speciation exhibits certain patterns and variations. Overall, most heavy metals (such as Cd, Cr, Cu, Ni, Pb, and Zn) primarily exist in the form of residues across all samples, with proportions generally exceeding 85%. This indicates that these heavy metals predominantly exist in manure as stable mineral lattice structures, exhibiting low bioavailability and mobility, and posing relatively minor environmental risks. However, the distribution of As speciation varied significantly across different samples. In pig farm samples (GJPM, LSCPM, GPPM), the proportion of residual As remained high (particularly in GJPM at 75.46%), while in poultry farm samples (PJCM, YXCM), the proportions of acid-exchangeable and oxidizable As significantly increased. Notably, oxidizable As constituted 67.01% of total As in YXCM, indicating its potential activation and release under oxidizing conditions, posing a high potential environmental risk [24]. This discrepancy may be related to the types and quantities of feed additives used during the rearing process, particularly the common addition of As-containing growth promoters in chicken feed, which leads to the accumulation of bioavailable As in manure [25]. In terms of farming scale, larger-scale farms (YXCM, GPPM) exhibited relatively higher levels of acid-exchangeable heavy metals. For instance, the proportion of acid-exchangeable Cd and Pb in YXCM was higher than in other samples, suggesting that large-scale intensive farming may lead to the accumulation of certain active forms of heavy metals due to standardized feed and centralized manure discharge [3]. In contrast, small-scale farm (GJPM) exhibited higher proportions of residual heavy metals, which were more stable. This may be related to their farming practices, feed sources, or manure storage conditions [23]. In summary, the environmental risks posed by heavy metals in manure in Liuyang City are generally manageable. However, As exhibits higher bioavailability in chicken manure, warranting particular attention. Expanding farming scales may increase the bioavailability of certain heavy metals. It is recommended to strengthen oversight of As-containing feed additives in large-scale farms and promote the harmless treatment and resource utilization of manure to mitigate potential ecological and health risks associated with heavy metals [24].

### 3.3. Correlations Between Antibiotics and Heavy Metals

**Figure 4:**
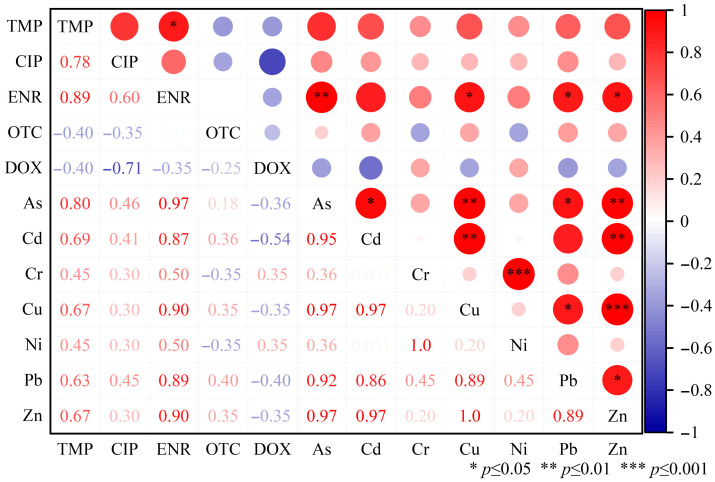
Correlation analysis of heavy metals and antibiotics in animal manure.

Spearman’s correlation analysis of heavy metal and antibiotic levels in manure samples (as shown in Figure 4) revealed a significant correlation between antibiotics and heavy metals, suggesting their coexistence may pose potential risks to the environment and public health. TMP, CIP, and ENR exhibited a high positive correlation, with correlation coefficients of 0.98, 0.99, and 0.90, respectively. This suggests that these antibiotics may coexist in manure due to concurrent use during farming or similar metabolic pathways [26]. OTC and DOX also exhibited a high correlation (0.93), further supporting the coexistence of tetracycline antibiotics in manure. Significant correlations were also observed among the heavy metals, such as As, Cd, Cr, Cu, Ni, Pb, and Zn. The correlation coefficient between As and Cd is 0.90, indicating that these two heavy metals may co-accumulate in manure, possibly due to their similar chemical behavior or sources in the environment. The correlation coefficient between Cu and Zn is 0.97, also reflecting that these two heavy metals may be used together in feed additives [27]. It is noteworthy that significant correlations also exist between antibiotics and certain heavy metals. The correlation coefficient between As and TMP was 0.80, while that with CIP was 0.78, potentially indicating the co-occurrence of As with these antibiotics in manure. The correlation coefficient between Cd and ENR was 0.87, possibly reflecting the joint presence of Cd and sulfonamide antibiotics in manure. These correlations may have implications for environmental and public health. The coexistence of antibiotics and heavy metals may increase the risk of spreading antibiotic resistance genes, while also posing a threat to human health through the food chain [24]. Furthermore, the coexistence of heavy metals and antibiotics may affect the degradation and transformation of antibiotics, thereby influencing their environmental behavior and persistence [28].

The Spearman correlation analysis results reveal complex interactions between antibiotics and heavy metals in livestock manure. These findings hold significant implications for developing effective manure management strategies and mitigating environmental risks. Future research should further explore the underlying mechanisms of these correlations and assess their long-term impacts on ecosystems and human health.

### 3.4. Manure Microbial Community Composition and Functional Prediction Analysis

PCoA based on bacterial community composition was performed to further explore the differences and similarities among the microbial communities in the five manure samples. As shown in Figure S1), the samples clustered distinctly according to their groups. Notably, PJCM samples formed a separate cluster along the PCoA2 axis, indicating a unique microbial community structure compared to other groups. YXCM and GPPM samples clustered separately on the left side of the plot, reflecting differences in their community compositions. LSCPM and GJPM samples were positioned closely together, suggesting similar microbial community structures between these two pig manure groups. Together, the first two principal coordinates explained approximately 72.77% of the total variation, highlighting significant differences in microbial community composition across different manure types and farming environments.

**Figure 5:**
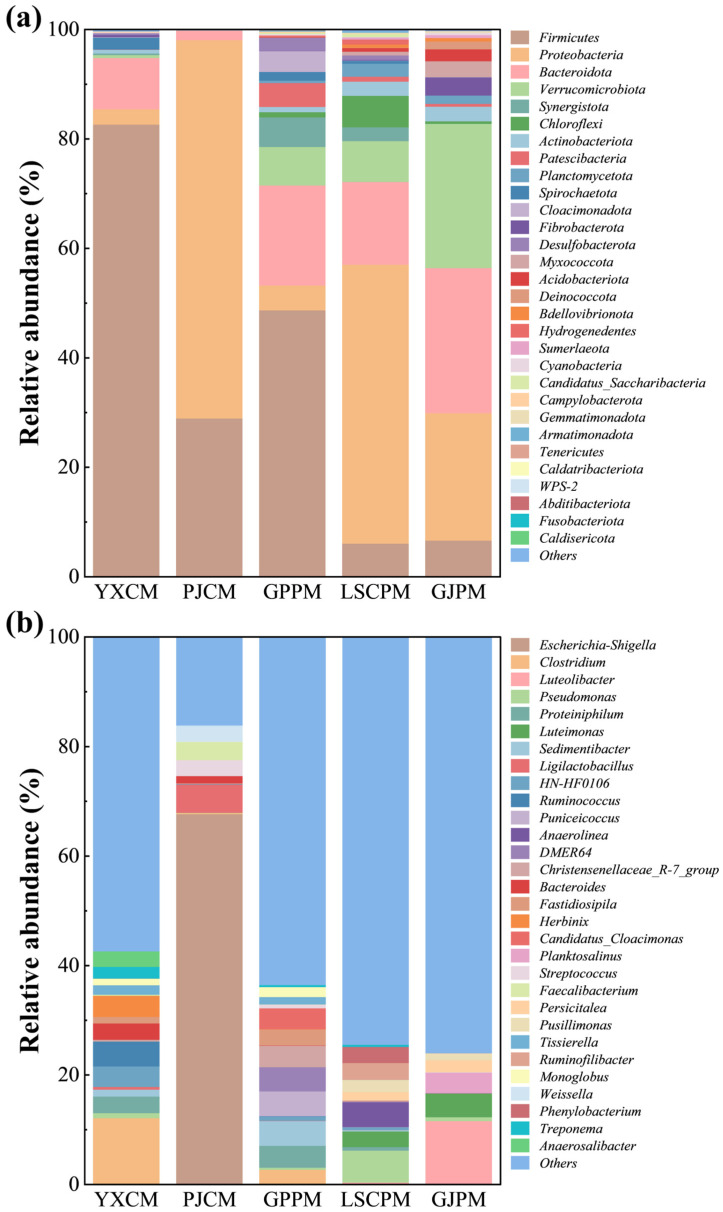
Relative abundance of microbial composition structure at the phylum (**a**) and genus (**b**) level.

Based on 16S rDNA sequencing results, a comparative analysis of microbial composition at the phylum and genus levels were conducted for five manure samples. The results revealed significant differences in microbial community structure among the samples. As shown in Figure 5a, in chicken manure samples YXCM and PJCM, *Firmicutes* and *Proteobacteria* were the dominant phyla. In YXCM, *Firmicutes* constituted a high proportion of 82.59%, while *Proteobacteria* accounted for 69.14% in PJCM, indicating differences in their major bacterial compositions. Pig manure samples (GPPM, LSCPM, and GJPM) exhibited more diverse community structures. In GPPM, *Firmicutes* and *Proteobacteria* accounted for 48.63% and 4.54%, respectively, while *Proteobacteria* and *Bacteroidota* dominated in LSCPM and GJPM at 50.99% and 15.14%. Additionally, the relative abundance of phyla such as *Verrucomicrobiota*, *Synergistota*, and *Chloroflexi* was significantly higher in pig manure samples compared to chicken manure samples, reflecting greater microbial diversity in pig manure communities. As shown in Figure 5b, the *Escherichia-Shigella* genus dominates the chicken manure sample PJCM with an abundance as high as 67.61%, indicating its predominant status in this sample. This may be related to the farming environment and antibiotic use, suggesting a potential risk of pathogenic bacteria. Another chicken manure sample, YXCM, exhibits a more diverse microbial community structure, with dominant genera including *Clostridium*, *Ruminococcus*, and *Herbinix*, indicating higher community complexity. In pig manure samples, GPPM showed abundant genera such as *Puniceicoccus*, *Sedimentibacter*, and *DMER64*, while LSCPM featured prominent *Pseudomonas* and *Anaerolinea*. GJPM was characterized by genera like *Luteolibacter* and *Luteimonas*. The microbial communities in manure are dominated by *Firmicutes* and *Proteobacteria* phyla. However, significant differences in community structure at both the phylum and genus levels are observed across different animal species and farming environments. The enrichment of certain potentially pathogenic genera in chicken manure indicates a need for public health vigilance, while the high diversity in pig manure may enhance its capacity for degrading antibiotics and heavy metal pollutants and for adapting to such contaminants [2,29]. Further correlation analysis (Figure S2) revealed significant associations between microbial genera and concentrations of heavy metals and antibiotics in the environment. Genera such as *Luteolibacter*, *Luteimonas*, and *Planktosalinus* showed significant positive correlations with antibiotics TMP and ENR, suggesting these microorganisms may possess strong pollutant tolerance and accumulate in contaminated environments. Conversely, genera such as *Clostridium*, *Herbinix*, and *Tissierella* showed significant negative correlations with multiple heavy metals and antibiotics, reflecting their sensitivity or suppression by pollutants. These findings indicate that heavy metals and antibiotics, acting as environmental selection pressures, are key drivers of microbial community structure changes, with distinct microbial genera exhibiting markedly different responses to pollutants.

**Figure 6:**
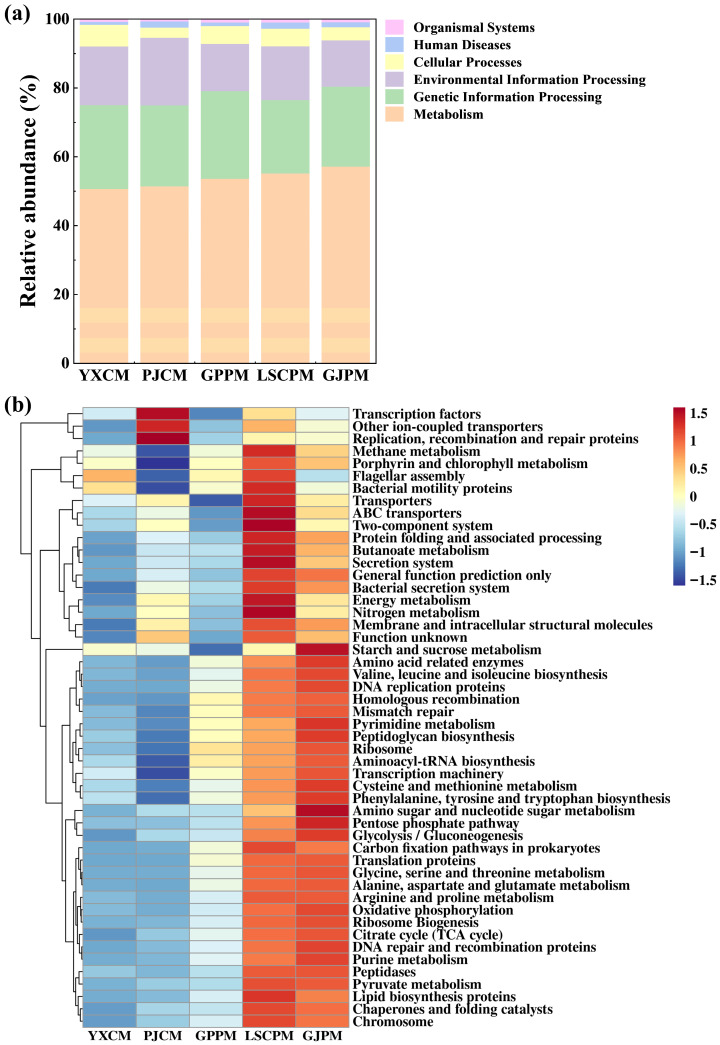
Functional prediction pathways at level 1 (**a**) and level 3 (**b**).

Functional prediction results based on 16S rDNA sequencing data reveal significant structural differences and potential ecological functions across various functional levels within manure microbial communities. Level 1 functional prediction (as shown in Figure 6a) indicates that the primary functions of microbial communities are concentrated in three major categories: metabolism, genetic information processing, and environmental information processing. Metabolic functions accounted for the largest proportion, approximately 50% to 60%, reflecting the microbial community’s robust capacity for material conversion and energy metabolism. Genetic information processing functions constituted about 20% to 25%, indicating high microbial activity in critical life processes such as gene expression, replication, and repair. The stable presence of environmental information processing functions demonstrated the microbes’ ability to perceive and respond to external environmental signals [30]. Level 2 functional predictions further refined these major categories. As shown in Figure S3, pig manure samples exhibited high activity in multiple metabolic pathways, including amino acid metabolism, carbohydrate metabolism, energy metabolism, and metabolism of cofactors and vitamins. This suggests the microbial community possesses strong potential for organic matter decomposition and nutrient conversion. Additionally, significant enrichment in xenobiotics biodegradation and metabolism functions indicates robust capabilities for transforming pollutants like antibiotics and heavy metals, thereby mitigating environmental risks [31]. In contrast, chicken manure samples exhibited lower functional abundance in these pathways, reflecting relatively weaker microbial community activity. Level 3 functional predictions revealed more specific metabolic and physiological processes. As shown in Figure 6b, pig manure samples exhibited high abundance in energy metabolism pathways such as glycolysis / gluconeogenesis, pentose phosphate pathway, oxidative phosphorylation, and citrate cycle (TCA cycle), demonstrating robust energy acquisition and material synthesis capabilities. Functions related to protein synthesis (e.g., ribosome, translation proteins) and genetic material maintenance (DNA replication proteins and DNA repair and recombination proteins) were also significantly enhanced in pig manure. This indicates that the microbial community is in a highly metabolically active state and possesses strong environmental adaptation and repair capabilities [32]. The activity of signal transduction and transport proteins further supports the microbial response mechanisms to environmental stressors. Collectively, the functional diversity of livestock manure microbial communities is closely linked to their structural characteristics. The pig manure microbial community exhibits high metabolic activity and environmental adaptability, facilitating the biodegradation and transformation of pollutants such as antibiotics and heavy metals [33,34]. In contrast, the enrichment of potentially pathogenic bacterial genera and lower functional activity in chicken manure suggest public health risks, necessitating enhanced environmental monitoring and management.

### 3.5. Risk Assessment of Antibiotics and Heavy Metals

#### 3.5.1. Ecological and Health Risk Assessment Based on Total Antibiotics

**Figure 7:**
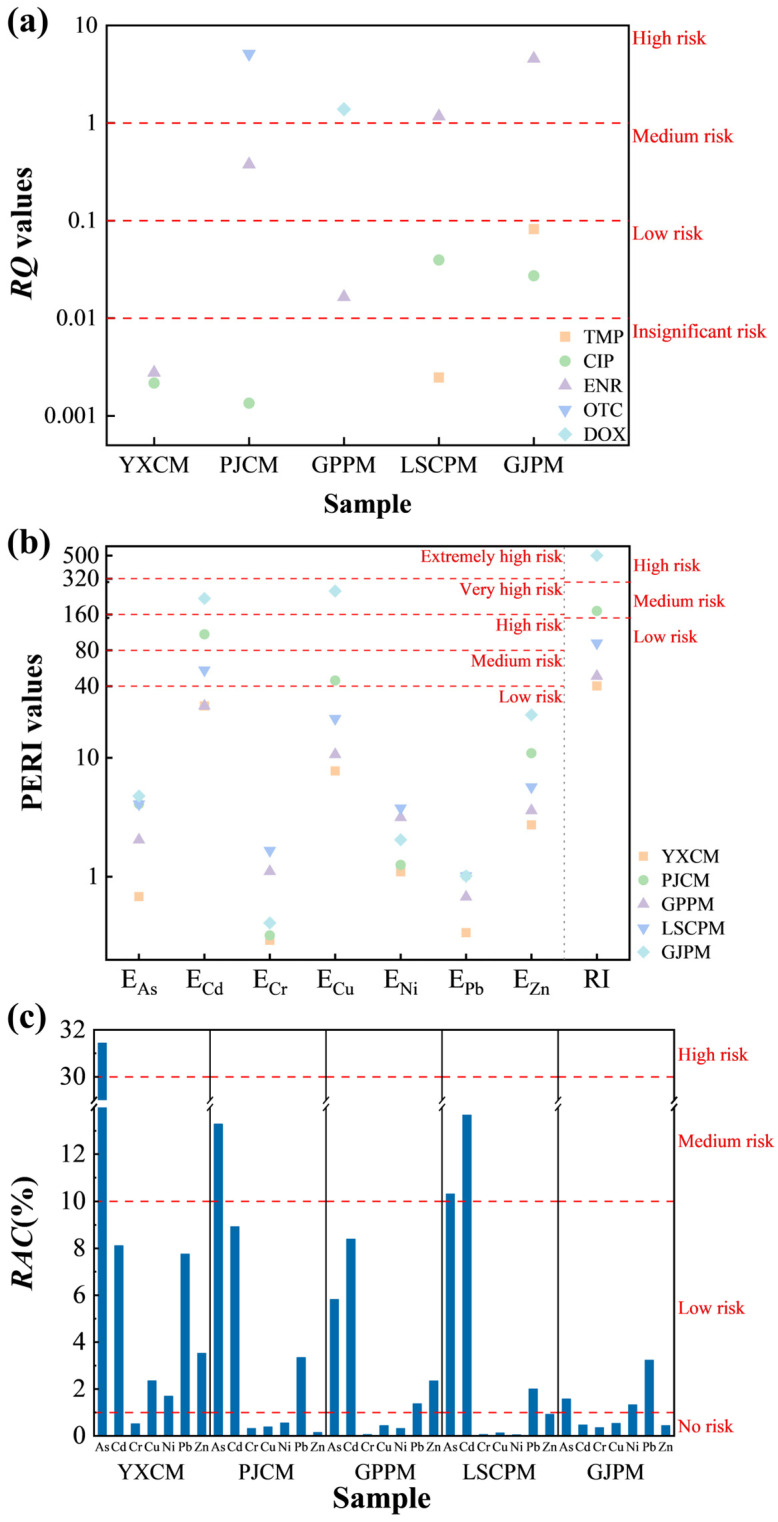
The values of RQ (**a**), $E_{i}$ & RI (**b**) and RAC (**c**) in manure.

Risk quotients (RQ) for antibiotic residues in manure samples from various sources were assessed, revealing the complexity and variability of ecological risks associated with antibiotic contamination in such manure. The assessment results (as shown in Figure 7a) reveal a significantly skewed distribution of risks. All target antibiotics in the PJCM samples exhibited RQ values below 0.01, indicating an insignificant risk, whereas other manure samples contained antibiotics posing medium to high risks. Among these, ENR exhibits high risk in both LSCPM (RQ = 1.170) and GJPM (RQ = 4.576), and medium risk in PJCM (RQ = 0.377); DOX was high-risk in GPPM (RQ = 1.387), while OTC showed extremely high risk in PJCM (RQ = 5.116). Other antibiotics such as CIP and TMP mostly exhibited RQ values within low risk or insignificant risk. Further analysis revealed that ecological risks do not exhibit a simple linear relationship with farm scale. High-risk residues were detected in both large-scale farms and small-to-medium-sized farms, indicating that risks are not exclusive to smaller operations. Antibiotic management in large-scale intensive farming may also harbor deficiencies. The formation of this high-risk pattern is driven by multiple factors. The primary factor is the intensity and pattern of antibiotic use. As commonly used and high-volume broad-spectrum antimicrobials in livestock and poultry farming, ENR and OTC are frequently administered or used therapeutically. This leads directly to high initial residual concentrations in manure, forming the material basis for high risk [24]. The environmental behavior characteristics of antibiotics themselves play a crucial role. Both ENR and OTC are known for their high environmental persistence, tendency to absorb onto solid particles, and poor biodegradability. They are difficult to effectively remove during manure composting or storage, allowing them to enter the environment in highly active forms and continuously exert ecotoxicological pressure [35]. Conversely, certain lower-risk antibiotics may be more readily transformed or degraded in the environment due to their chemical structure.

Antibiotics posing high ecological risks, such as ENR and OTC, can enter soil-crop systems through manure application. They may migrate to aquatic environments via bioaccumulation and transfer within the food chain, or through pathways like surface runoff and soil leaching. This creates multiple potential routes of human exposure, significantly increasing the risk of long-term, low-dose exposure in populations [36]. Long-term exposure to such a complex contaminated environment containing residues of multiple antibiotics may not only cause direct health damage due to the antibiotics’ inherent toxicity, but more alarmingly, this persistent environmental exposure exerts powerful selective pressure. This significantly accelerates the generation, enrichment, and horizontal transfer of antibiotic resistance genes (ARGs) within environmental microorganisms and pathogenic bacteria [36]. ARGs and bacteria can further spread through environmental media and the food chain into human activity spheres, leading to reduced efficacy or even failure of commonly used clinical antibiotics. This poses a serious threat to public health security and undermines the effectiveness of infectious disease prevention and control efforts [37]. This issue of antibiotic resistance spreading due to environmental residues has become one of the major challenges facing global health today, owing to its hidden nature, persistence, and global reach.

#### 3.5.2. Assessment of Pollution Levels Based on Total Heavy Metals

The results for potential ecological risks of heavy metals (as shown in Figure 7b) indicate significant differences in RI values among different samples. Specifically, the GJPM sample (RI = 501.27) exhibits a high ecological risk level, the PJCM sample (RI = 171.26) shows medium ecological risk, while the remaining three samples (YXCM, GPPM, LSCPM) fall under the category of low ecological risk. Among numerous heavy metals, Cd stands as the primary ubiquitous risk factor, with its $E_{i}$ reaching high or very high risk levels in multiple samples. The pollution contribution and accumulation characteristics of Cu also warrant significant attention. Calculations reveal that the $E_{i}$ value for Cu in the GJPM sample is exceptionally high at 251.94, far exceeding other samples (ranging from 7.75 to 44.57) and significantly surpassing the threshold for very high ecological risk. This makes it the key driver behind the elevated overall risk in this sample. In pig farming, high doses of Cu (such as copper sulfate) are commonly added to feed as growth promoters, which directly leads to a significant increase in Cu content in manure [38]. The extremely high Cu risk presented by the GJPM sample may stem from its use of feed formulations with elevated Cu content. In contrast, larger-scale pig farms (such as GPPM and LSCPM) exhibit lower risks of Cu contamination in manure. This may be attributed to the advantages of large-scale operations in standardizing feed formulations, refining nutritional management, and implementing standardized manure management practices. These measures enable more effective control over excessive heavy metal supplementation and emissions [39]. Analysis of farming types and scales revealed that among chicken manure samples, the larger-scale YXCM exhibited a lower RI value than the smaller-scale PJCM. This discrepancy may be attributed to differences in feed sources, farming management practices, and manure treatment measures. Among the pig manure samples, GJPM, despite having the smallest farming scale, exhibited the highest ecological risk, with significantly higher $E_{i}$ values for Cd and Cu compared to other pig farms. This suggests that this farm may have unique characteristics related to feed additive usage, environmental background levels, or manure storage practices. Overall, the risk of heavy metal accumulation in pig manure samples is more pronounced than in chicken manure, particularly for Cd and Cu. This may be related to pigs’ absorption and metabolic characteristics of heavy metals, as well as commonly used feed formulations [25].

The ecological risks of heavy metals in manure are primarily driven by heavy metals such as Cd and Cu. Manure from small-scale farms, where management practices may be relatively lax, exhibits higher pollution risks, which is directly linked to their unique history of feed additive usage [39]. Therefore, to effectively prevent and control the environmental risks of heavy metals associated with the agricultural use of manure, it is essential not only to maintain vigilance against widespread Cd contamination but also to prioritize the standardized use of Cu additives in feed. It is recommended to establish targeted Cu content monitoring and risk management mechanisms for pig manure, particularly from small and medium-sized farms, to ensure the safe resource utilization of manure.

#### 3.5.3. Assessment of Pollution Levels Based on Heavy Metal Speciation

Based on the results of the RAC analysis for heavy metals in five manure samples (as shown in Figure 7c), the ecological risk of heavy metals in manure from different sources was systematically evaluated. Overall, the RAC values for different heavy metals across samples varied significantly, reflecting the complex characteristics of heavy metal bioavailability. Specifically, the RAC value for As in YXCM reached 31.44%, indicating a high-risk level, while most other heavy metals remained at low risk levels. In PJCM, As exhibited medium risk (13.28%), while other heavy metals posed lower risks. Among pig manure samples, LSCPM showed medium risk levels for both As and Cd (10.30% and 13.67%, respectively). RAC values for heavy metals in the remaining pig manure samples were mostly below 10%, falling into the low-risk or no-risk category. From the perspective of livestock type and farming scale, chicken manure samples exhibited higher overall ecological risks from heavy metals than pig manure samples, showing a positive correlation with farming scale. The larger-scale YXCM exhibits a particularly high risk for As, likely stemming from the cumulative effects of As-containing feed additives or environmental media during intensive farming. In contrast, the smaller-scale PJCM shows relatively lower As risk, indicating that increased farming scale may intensify the accumulation of bioavailable forms of certain heavy metals in manure. In contrast, no clear correlation was observed between RAC values in pig manure samples and farm scale: the largest-scale GPPM exhibited overall lower risks for all heavy metals, while medium-scale LSCPM showed medium risks for As and Cd, and the smallest-scale GJPM presented the lowest risks. This indicates that the bioavailability of heavy metals in pig manure is not only influenced by farm scale but may also be significantly regulated by multiple factors including feed composition, farming environment, manure management practices, and geographical origin [40].

The ecological risks of heavy metals in manure depend not only on their total quantities but are also closely related to their speciation and bioavailability. The relatively high risk of As in chicken manure warrants attention, particularly in large-scale farms where it should be prioritized for control. While the heavy metal risks in pig manure are comparatively lower, the accumulation of As and Cd in some samples indicates that local farming environments and feed safety still require vigilance [41]. The findings indicate that when assessing the environmental risks of heavy metals in manure, differentiated analysis should be conducted based on the species of livestock and poultry, the scale of farming operations, and the specific forms of heavy metals. This provides a scientific basis for the rational resource utilization and risk management of agricultural waste.

#### 3.5.4. Health Risk Assessment of Heavy Metals

##### Non-Carcinogenic Risk Analysis of Heavy Metals

**Figure 8:**
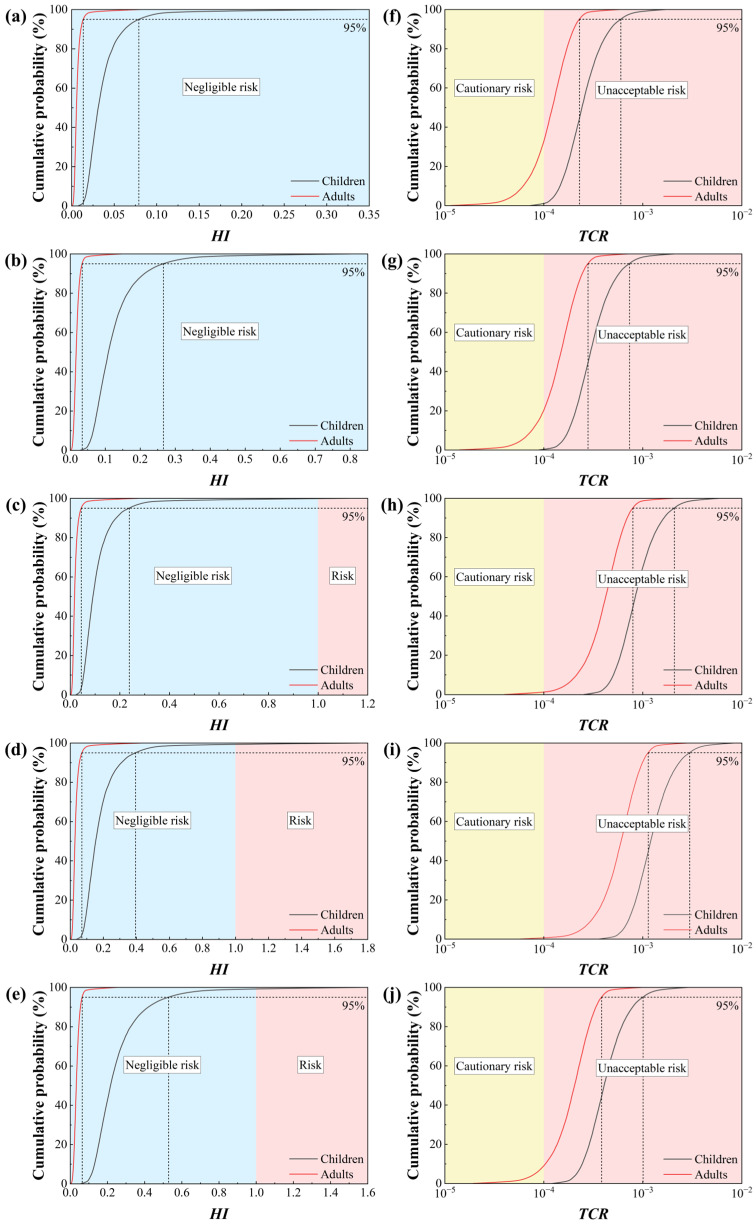
The values of HI in YXCM (**a**), PJCM (**b**), GPPM (**c**), LSCPM (**d**), GJPM (**e**), and the values of TCR in YXCM (**f**), PJCM (**g**), GPPM (**h**), LSCPM (**i**) and GJPM (**j**).

Using Monte Carlo simulation techniques, exposure risks from heavy metals in manure were assessed through high percentile and extreme value analysis of non-carcinogenic hazard indices (HI), as shown in Figure 8a–e. Focusing on the 95th percentile (reflecting higher-risk scenarios) and maximum values (representing extreme risk scenarios) reveals significant variations in risk characteristics across different livestock species and farming scales [16]. For adults, the maximum HI values and 95th percentile values for all samples were well below the safety threshold (HI < 1), indicating that even under extreme exposure assumptions, the non-carcinogenic risk is acceptable. However, the risk assessment results for the children revealed potential risks warranting attention. Specifically, in pig manure samples, LSCPM exhibited a maximum HI value of 1.750779 for children, exceeding the risk threshold. Its 95th percentile was 0.395030, which, while not exceeding the standard, was already at a relatively high level. This indicates that there is a probability of unacceptable non-carcinogenic risk occurring at this site, although the likelihood remains low. Among pig manure samples, the maximum HI values for children from both the largest-scale GPPM and smallest-scale GJPM farms did not exceed 1, yet their 95th percentile values reached 0.238194 and 0.528499, respectively. This phenomenon indicates that the heavy metal risk associated with pig manure does not exhibit a direct linear correlation with farm scale. Instead, it is more likely determined by specific factors unique to individual farms, such as feed formulations, manure management practices, and local environmental background levels. In contrast, chicken manure samples showed that the maximum HI values and 95th percentile values for children from both chicken farms did not exceed 1, indicating overall controllable risks. Notably, the larger-scale YXCM site exhibited significantly lower maximum HI (0.334756) and 95th percentile (0.079058) values compared to the smaller-scale PJCM site (maximum 0.799523, 95th percentile 0.266344). This suggests that large-scale, intensive chicken farms may more effectively suppress heavy metal accumulation and related exposure risks through the implementation of more standardized feed additive controls and more regulated manure treatment measures. Sensitivity analysis (as shown in Table S13) indicates that children exhibit high sensitivity to body weight (BW) and skin contact parameters (SL, SA), explaining from a physiological perspective why their risk levels are generally higher than those of adults [16,42].

Analysis of the maximum values and 95th percentile quantiles indicates that while the non-carcinogenic risks to children from heavy metals in manure fall within acceptable ranges under most scenarios, extreme risks exceeding thresholds may occur at specific locations (such as LSCPM) [42]. Risk management should prioritize pig farms, particularly small-to-medium-sized operations where management measures may be relatively weak. It is essential to strengthen heavy metal control across the entire chain, from feed sources to manure disposal, to reduce the likelihood of high-risk exposure incidents.

##### Analysis of the Carcinogenic Risks of Heavy Metals

Monte Carlo simulations were employed to assess the carcinogenic risk of heavy metals in manure from five farms, with a focus on their statistical distribution characteristics, particularly the risk thresholds reflected by the maximum value and the 95th percentile. The results (as shown in Figure 8f–j) indicate that the average TCR for the pediatric population exceeded the USEPA unacceptable risk threshold of 1 × 10^−4^ at all sampling sites. Risks were most pronounced at the large-scale pig farm (GPPM) and medium-scale pig farm (LSCPM), with pediatric TCR averages reaching 0.000992 and 0.001416 respectively. Even smaller-scale pig farms (GJPM) showed significantly elevated average TCR values for children (0.000479). In contrast, while the two chicken farms exhibited relatively lower risks, their average TCR values for children (YXCM: 0.000286; PJCM: 0.000349) still exceeded acceptable levels. The risk trends in adults were consistent with those in children, though the values were generally lower, with some sample averages at the upper limit of the acceptable range or slightly exceeding the threshold. This result clearly reveals that heavy metals in manure within the study area, particularly from pig farms, pose a non-negligible carcinogenic risk to the surrounding population, especially children [16,42]. Sensitivity analysis (as shown in Table S13) indicates that direct ingestion rate (Ring) and body weight (BW) are key parameters influencing TCR distribution. Particularly in pediatric populations, lower body weight combined with higher direct ingestion behavior significantly amplifies the probability of high-risk scenarios. This explains why risk thresholds for children are generally elevated and more likely to exceed acceptable limits [16,43].

Analysis combining farm scale and livestock type revealed that medium-scale farm LSCPM exhibited the highest 95th percentile values for adults and children TCR (0.001135 and 0.002975, respectively) and maximum values (0.002910 and 0.008481, respectively) among all samples, surpassing even larger-scale farm GPPM. This suggests that the key determinant of carcinogenic risk may not lie solely in the scale of farming operations, but is closely related to factors such as stocking density per unit area or under specific management systems, levels of heavy metal additives in feed, localized accumulation and disposal methods of manure, as well as specific environmental factors at sampling sites [24,25]. In contrast, for chicken manure samples, the larger-scale farm YXCM exhibited systematically lower mean TCR values, 95% corresponding values, and maximum values across both adults and children compared to the smaller-scale farm PJCM. This finding reveals that within poultry farming systems, the primary risk driver is not the scale of production but rather the quality and standardization of management practices. Large-scale farming operations often employ more scientific and controllable feed formulation systems, which may impose stricter restrictions on heavy metal additives and provide alternative solutions, thereby reducing heavy metal inputs at the source. Simultaneously, these facilities typically feature more comprehensive manure removal, collection, and centralized treatment systems, minimizing opportunities for heavy metals to linger and accumulate within the farming environment and lowering the potential for localized exposure [2,39,44].

In summary, the carcinogenic risk posed by heavy metals in manure depends not only on the scale of farming operations and animal species but is also closely linked to the behavioral and physiological characteristics of exposed populations. Particularly under extreme scenarios, the associated risks cannot be overlooked. It is recommended that risk management prioritize differentiated controls based on farming types and pay special attention to protecting children from exposure to mitigate potential health risks.

## 4. Conclusions

This study systematically analyzed the pollution characteristics and combined risks of antibiotics and heavy metals in manure collected from five livestock and poultry farms of varying scales in Liuyang City, Hunan Province. Widespread antibiotic contamination was observed, with fluoroquinolones (such as ENR and CIP) significantly higher in pig manure than in chicken manure, while tetracycline and macrolide distributions showed site-specific patterns. Heavy metal contamination was mainly characterized by Cu and Zn accumulation, with certain samples from small-scale farms exceeding standard limits. Most heavy metals existed in stable forms, though As in chicken manure displayed high bioavailability. Significant correlations were found among multiple antibiotics, heavy metals, and specific antibiotic–heavy metal pairs (such as As–TMP and As/Cd–ENR), suggesting combined sources and potential synergistic effects. Ecological risk assessment indicated medium-to-high risks from antibiotics such as ENR and OTC, while Cd and Cu were the primary heavy metal risk factors, especially in small-scale pig farms. Functional prediction of 16S rDNA data indicates that the microbial community in pig manure exhibits high metabolic activity, demonstrating strong environmental adaptability and pollutant degradation capabilities. In contrast, chicken manure harbors higher abundances of potential pathogens, posing public health risks. Health risk simulations revealed that children face higher non-carcinogenic and carcinogenic risks from heavy metals than adults, with some pig farm samples exceeding acceptable risk thresholds. Overall, pollution levels did not correlate linearly with farm scale but were influenced by management practices, feed additives, and exposure factors. The findings provide a scientific basis for regional livestock pollution control and safe manure utilization, highlighting the need for stricter regulation of feed additives, prioritized monitoring of Cu, Zn, and bioavailable As, and protective measures for vulnerable populations. Further large-scale and long-term monitoring is recommended to clarify the behavior and effects of combined pollutants across environmental media.

## Data Availability

The original data presented in this study are included in this article; further inquiries can be directed to the corresponding author.

## Supplementary Materials

The following supporting information can be downloaded at: https://www.mdpi.com/article/10.3390/toxics14030201/s1, Figure S1: PCoA plots of beta diversity of microbial communities in different leaching agents based on Bray–Curtis distances; Figure S2: Correlation analysis between the top 30 microbial genera and the concentrations of heavy metals and antibiotics; Figure S3: Functional prediction pathways at level 2; Table S1: Sample collection information; Table S2: Detailed parameters for LC-MS/MS; Table S3: Gradient elution program; Table S4: Microwave digestion procedure; Table S5: PCR reaction system; Table S6: PCR reactions parameters; Table S7: Heavy metal background values; Table S8: Potential ecological risk index level classification; Table S9: Risk assessment code level classification; Table S10: Parameter reference values; Table S11: RfD and SF for different exposure routes; Table S12: Concentrations of major antibiotics (μg/kg) and heavy metals (mg/kg); Table S13: Sensitivity analysis of HI and TCR [14,15,17,45].
