# Supplementary material for: Environmental and Health Risk Assessments of Antibiotics and Heavy Metals in Manure in Liuyang City
Source: Toxics. 2026 Feb 27;14(3):201. doi: 10.3390/toxics14030201 (PMC13030225; doi:10.3390/toxics14030201)
Supplement: Supplementary file 1 [file toxics-14-00201-s001.zip › toxics-4142841-supplementary.pdf]

Environmental and Health Risk Assessments of Antibiotics and Heavy Metals in Manure in Liuyang City

Yuli Jiang [1,2], Ziwen Guo [1,2], Manjun Miao [1,2], Xueduan Liu [1,2], Luhua Jiang [1,2*]

[1] School of Minerals Processing and Bioengineering, Central South University, Changsha 410083, China

[2] Key Laboratory of Biometallurgy of Ministry of Education, Central South University, Changsha 410083, China

* Correspondence: jiangluhua@csu.edu.cn (LH Jiang)

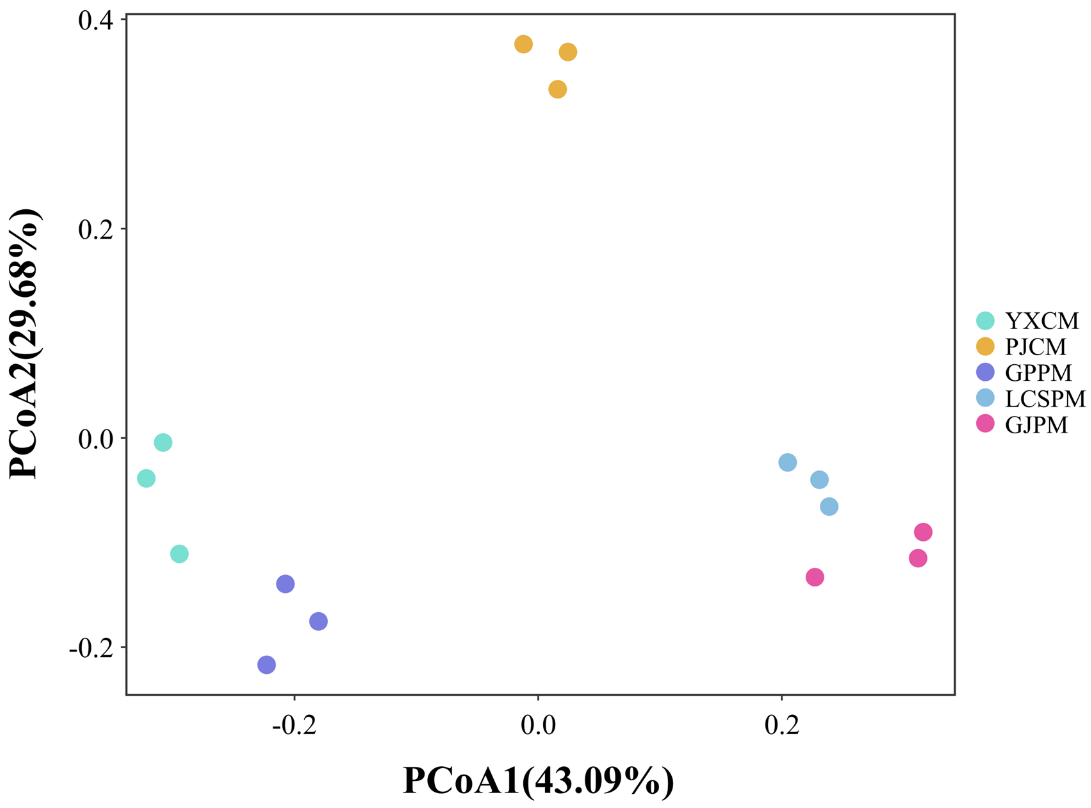

Figure S1. PCoA plots of beta diversity of microbial communities in different leaching agents based on Bray-Curtis distances.

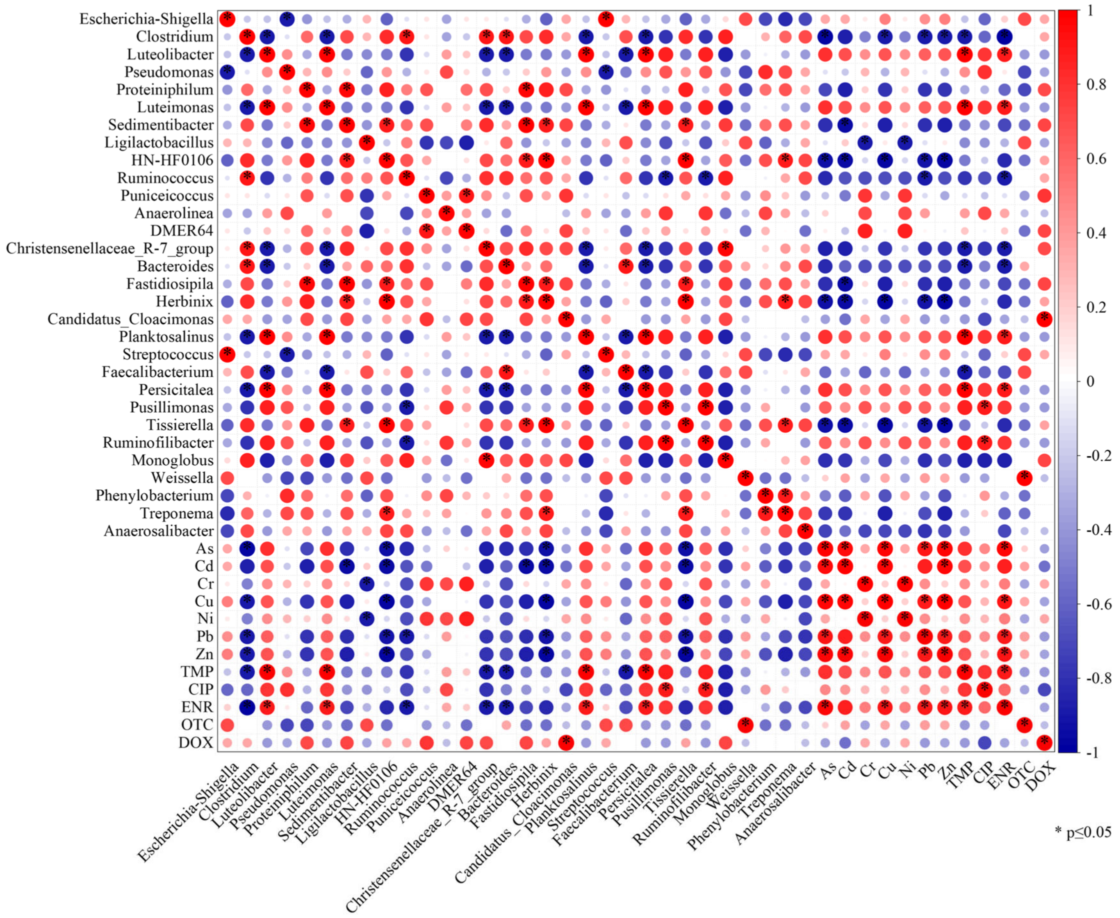

Figure S2. Correlation analysis between the top 30 microbial genera and the concentrations of heavy metals and antibiotics.

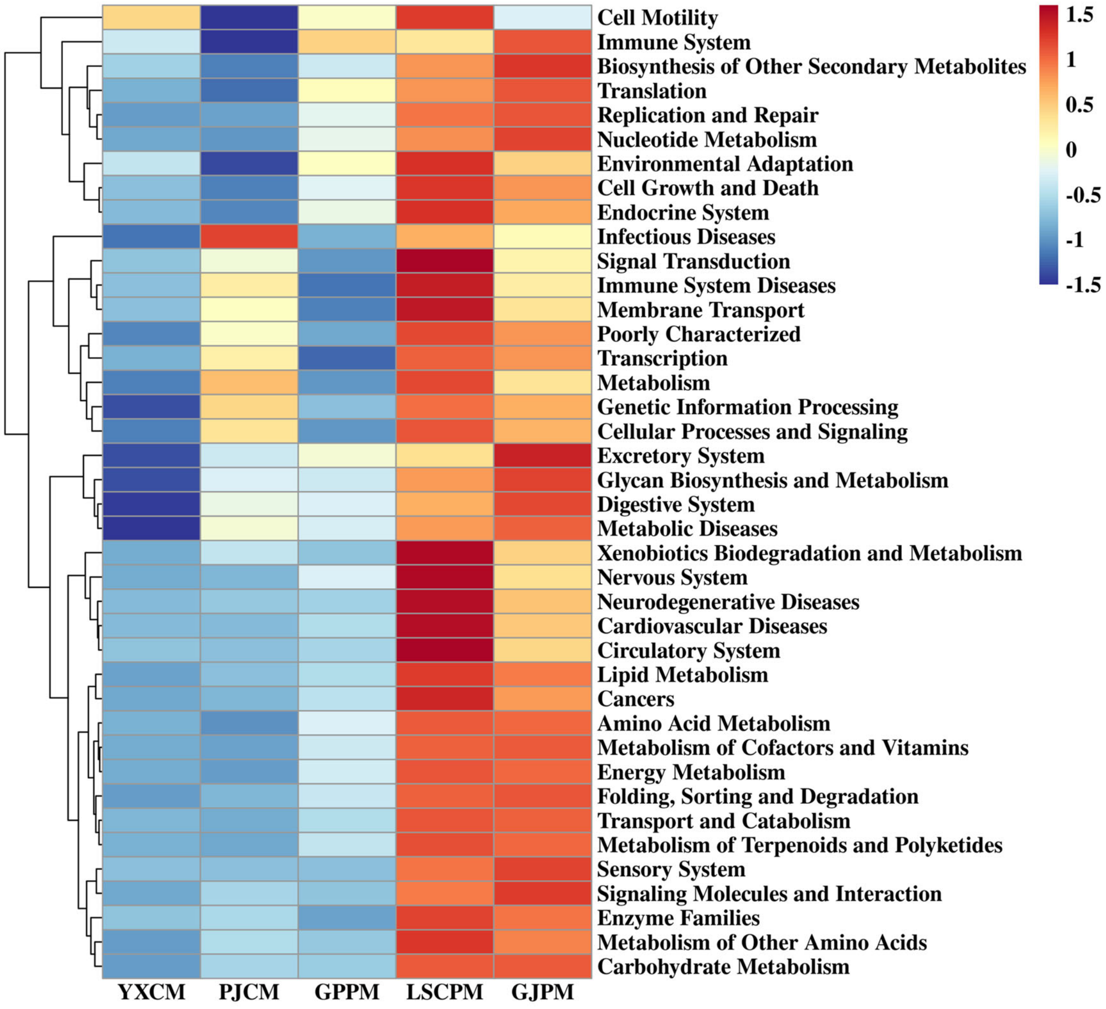

Figure S3. Functional prediction pathways at level 2.

Table S1 Sample collection information

Sample number	Sampling point coordinates	Types	Scale
YXCM	113.82°N,28.32°E	Chicken	10000
PJCM	113.40°N,27.98°E	Chicken	5000
GPPM	113.80°N,28.20°E	Pig	3000
LSCPM	113.78°N,28.08°E	Pig	1000
GJPM	113.46°N,28.07°E	Pig	500

Table S2 Detailed parameters for LC-MS/MS

Parameters	Conditions
Flow velocity	0.4 mL/min
Column temperature	35 ℃
Sample volume	5 μL
Mobile phase A	0.1% formic acid solution
Mobile phase B	0.1% formic acid-methanol/acetonitrile (V/V=1:1)
Elution procedure	Gradient elution
Ionization mode	ESI
DL temperature	250 ℃
Nebulizer air flow rate	3.00 L/min
Dry air flow rate	10.00 L/min

Table S3 Gradient elution program

Time (min)	Mobile phase A (%)	Mobile phase B (%)
2	70	30
5	60	40
7	5	95
8.5	5	95

Table S4 Microwave digestion procedure

Heat-up time	Decomposition temperature	Hold time
10 min	Room temperature → 120℃	5 min
5 min	120℃ → 160℃	5 min
5 min	160℃ → 190℃	25 min

Table S5 PCR reaction system

PCR reaction components	PCR reaction volume
Phusion® Hot Start Flex 2X Master Mixart Version	12.5 ul
Forward Primer (1μM)	2.5 ul
Reverse Primer (1μM)	2.5 ul
Template DNA	50 ng
Add ddH_2O to	25 ul

Table S6 PCR reactions parameters

PCR reaction components	PCR reaction time	Cycle
98 ℃	30 s	/
98 ℃	10 s	
50 ℃	30 s	32 cycles
72 ℃	45 s	
72 ℃	10 min	/

Table S7 Heavy metal background values[1]

Heavy metals	Background values (mg/kg)
As	14.7
Cd	0.11
Cr	68.6
Cu	25.8
Ni	31.8
Pb	29.6
Zn	91.6

Table S8 Potential ecological risk index level classification[2]

E_i	RI	Potential ecological risk level
$E_i < 40$	$RI < 150$	Low ecological risk
$40 \leq E_i < 80$	$150 \leq RI < 300$	Medium ecological risk
$80 \leq E_i < 160$	$300 \leq RI < 600$	High ecological risk
$160 \leq E_i < 320$	$600 \leq RI$	Very high ecological risk
$320 \leq E_i$	/	Extremely high ecological risk

Table S9 Risk assessment code level classification[2, 3]

RAC	Level
$RAC < 1\%$	No risk
$1\% \leq RAC < 10\%$	Low risk
$10\% \leq RAC < 30\%$	Medium risk
$30\% \leq RAC < 50\%$	High risk
$50\% \leq RAC < 70\%$	Extreme risk

Table S10 Parameter reference values[4]

Parameter	Description	Unit	Types and Values	
			Adults	Children
EF	Exposure frequency	d/a	TRI (180,345,365)	TRI (180,345,365)
R_{ing}	Ingestion rate	mg/d	TRI (4,30,52)	TRI (66,103,161)
SL	Skin adherence factor	mg/cm^2	LN (0.49,0.54)	LN (0.65,1.2)
R_{inh}	Inhalation rate	m^3/d	LN (9.01,1.26)	LN (7.71,1.27)
ED	Exposure duration	a	24	6
PEF	Particle emission factor	m^3/kg	1.36×10^9	1.36×10^9
BW	Average bodyweight	kg	LN (61.9,11.31)	TRI (5.25,29.3,56.8)
AT	Average exposure time	d	ED×365 (non-carcinogenic); 70×365 (carcinogenic)	ED×365 (non-carcinogenic); 70×365 (carcinogenic)
SA	Exposed skin area	cm^2	TRI (760,1530,4220)	TRI (430,860,2160)
ABF	Dermal absorption factor	-	0.001 (non-carcinogenic); 0.01 (carcinogenic)	0.001 (non-carcinogenic); 0.01 (carcinogenic)

Table S11 RfD and SF for different exposure routes[4]

HMs	RfD			SF		
	Ingestion	Dermal contact	Inhalation	Ingestion	Dermal contact	Inhalation
As	3.00×10^{-4}	1.23×10^{-4}	1.23×10^{-4}	1.5	3.66	15.1
Cd	1.00×10^{-3}	1.00×10^{-5}	1.00×10^{-5}	6.1	—	6.3
Cr	3.00×10^{-3}	6.00×10^{-5}	2.86×10^{-5}	5.00×10^{-1}	20	42
Cu	4.00×10^{-2}	1.20×10^{-2}	4.02×10^{-2}	—	—	—
Ni	2.00×10^{-2}	5.40×10^{-3}	2.06×10^{-2}	1.7	42.5	8.40×10^{-1}
Pb	3.50×10^{-3}	5.25×10^{-4}	3.52×10^{-3}	8.50×10^{-3}	—	4.20×10^{-2}
Zn	3.00×10^{-1}	6.00×10^{-2}	3.00×10^{-1}	—	—	—

Table S12 Concentrations of major antibiotics (μg/kg) and heavy metals (mg/kg)

	YXCM	PJCM	GPPM	LSCPM	GJPM
TMP	ND^{b}	ND^{b}	ND^{b}	$0.919±0.01^{b}$	$30.449±6.34^{a}$
CIP	$3.756±0.25^{c}$	$2.338±0.51^{c}$	ND^{c}	$68.576±7.71^{a}$	$47.172±1.8^{b}$
ENR	$0.277±0.06^{d}$	$37.743±1.12^{c}$	$1.653±0.03^{d}$	$117.032±15.78^{b}$	$457.568±18.35^{a}$
OTC	ND^{b}	$824.665±53.57^{a}$	ND^{b}	ND^{b}	ND^{b}
DOX	ND^{b}	ND^{b}	$163.075±6.74^{a}$	ND^{b}	ND^{b}
As	$1±0.05^{b}$	$6±1.04^{a}$	$3±0.58^{b}$	$6±0.89^{a}$	$7±0.16^{a}$
Cd	$0.1±0.02^{d}$	$0.4±0.04^{b}$	$0.1±0.01^{d}$	$0.2±0.02^{c}$	$0.8±0.01^{a}$
Cr	$10±1.11^{c}$	$11±1.56^{c}$	$38±1.04^{b}$	$57±3.15^{a}$	$14±1.63^{c}$
Cu	$40±3.47^{b}$	$230±12.78^{b}$	55±4.45	$110±7.92^{b}$	$1300±171.12^{a}$
Ni	$7±0.44^{d}$	$8±1.28^{d}$	$20±1.25^{b}$	$24±1.2^{a}$	$13±1.47^{c}$
Pb	$2±0.03^{c}$	$6±0.62^{a}$	$4±0.78^{b}$	$6±0.54^{a}$	$6±0.24^{a}$
Zn	$250±12.06^{c}$	$1000±39.79^{b}$	$330±25.78^{c}$	$520±111.53^{c}$	$2100±249.36^{a}$

Table S13 Sensitivity analysis of HI and TCR

		Parameter	YXCM	PJCM	GPPM	LSCPM	GJPM
HI	Adults	EF	0.2897	0.3194	0.2798	0.2855	0.3227
		R_{ing}	0.4787	0.6438	0.4354	0.4596	0.6752
		SL	0.6243	0.4240	0.6659	0.6432	0.3761
		R_{inh}	-0.0048	-0.0064	-0.0043	-0.0046	-0.0065
		BW	-0.3688	-0.4056	-0.3565	-0.3635	-0.4102
		SA	0.2342	0.1602	0.2498	0.2413	0.1427
	Children	EF	0.0021	0.0014	0.0024	0.0023	0.0013
		R_{ing}	0.0018	0.0020	0.0018	0.0018	0.0020
		SL	0.0097	0.0079	0.0104	0.0100	0.0076
		R_{inh}	0.0049	0.0048	0.0048	0.0049	0.0049
		BW	-0.0279	-0.0272	-0.0278	-0.0279	-0.0268
		SA	-0.0080	-0.0088	-0.0080	-0.0079	-0.0089
TCR	Adults	EF	-0.0018	-0.0018	-0.0018	-0.0018	-0.0018
		R_{ing}	0.0003	0.0003	0.0003	0.0003	0.0003
		SL	0.0145	0.0145	0.0145	0.0145	0.0145
		R_{inh}	0.0049	0.0049	0.0049	0.0049	0.0049
		BW	-0.0262	-0.0262	-0.0262	-0.0262	-0.0262
		SA	0.0135	0.0135	0.0135	0.0135	0.0135
	Children	EF	0.3269	0.3269	0.3269	0.3269	0.3269
		R_{ing}	0.4046	0.4046	0.4046	0.4046	0.4046
		SL	0.0004	0.0004	0.0004	0.0004	0.0004
		R_{inh}	-0.0038	-0.0038	-0.0038	-0.0038	-0.0038
		BW	-0.8324	-0.8324	-0.8324	-0.8324	-0.8324
		SA	0.0020	0.0020	0.0020	0.0020	0.0020

References

1 Wei, F.S.; Chen, J.S.; Wu, Y.Y.; Zheng, C.J. Study on Background Values of Soil Environmental Elements in China. Environ. Sci. 1991, 04, 12-19+94. https://doi.org/10.13227/j.hjkx.1991.04.005.

2 Verma, A.; Yadav, S.; Kumar, R. Geochemical fractionation, bioavailability, ecological and human health risk assessment of metals in topsoils of an emerging industrial cluster near New Delhi. *Environ. Geochem. Health* **2023**, *45* (12), 9041-9066. DOI: https://doi.org/10.1007/s10653-023-01536-5.

3 Sungur, A.; İşler, M. Geochemical fractionation, source identification and risk assessments for trace metals in agricultural soils adjacent to a city center (Çanakkale, NW Turkey). *Environ. Earth Sci.* **2021**, *80* (8), 299. DOI: https://doi.org/10.1007/s12665-021-09611-9.

4 Huang, J. L.; Wu, Y. Y.; Sun, J. X.; Li, X.; Geng, X. L.; Zhao, M. L.; Sun, T.; Fan, Z. Q. Health risk assessment of heavy metal(loid)s in park soils of the largest megacity in China by using Monte Carlo simulation coupled with Positive matrix factorization model. *J. Hazard. Mater.* **2021**, *415*. DOI: https://doi.org/10.1016/j.jhazmat.2021.125629.
